# Koschei the immortal and anti-aging drugs

**DOI:** 10.1038/cddis.2014.520

**Published:** 2014-12-04

**Authors:** M V Blagosklonny

**Affiliations:** 1 Department of Cell Stress Biology, Roswell Park Cancer Institute, BLSC, L3-312, Elm and Carlton Streets, Buffalo, NY, USA

## Abstract

In Slavic folklore, Koschei the Immortal was bony, thin and lean. Was his condition caused by severe calorie restriction (CR)? CR deactivates the target of rapamycin pathway and slows down aging. But the life-extending effect of severe CR is limited by starvation. What if Koschei's anti-aging formula included rapamycin? And was rapamycin (or another rapalog) combined with commonly available drugs such as metformin, aspirin, propranolol, angiotensin II receptor blockers and angiotensin-converting enzyme inhibitors.

## Facts

- Calorie restriction deactivates mTOR and increases life span
- Rapamycin prevents obesity and extends life span
- In fairy tales, long-lived heroes were lean, slim and bony

## Open Questions

- Were their leanness and longevity due to genetic inhibition of mTOR?
- Can leanness plus longevity be achieved by rapamycin?
- How to combine five clinically available anti-aging drugs with calorie restriction?

**Figure 1:**
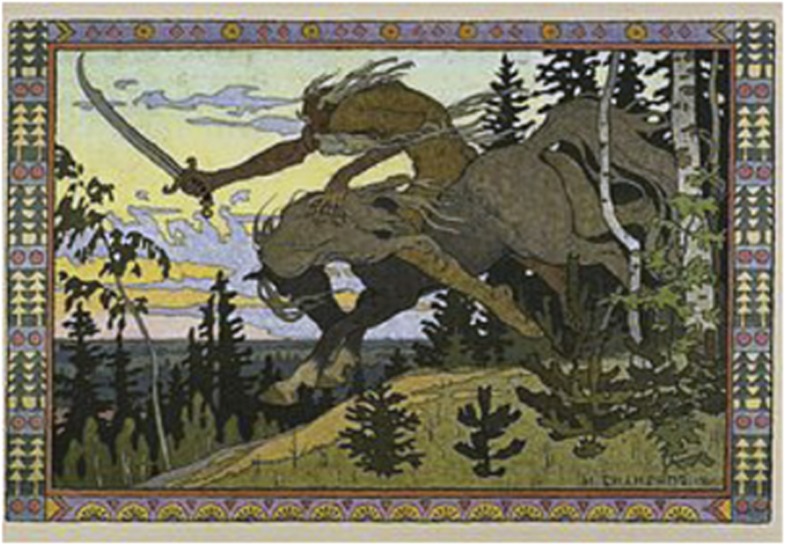
Koshchey the Deathless by Ivan Bilibin, 1901

Koschei the deathless (a villain in Russian, Polish and Ukrainian fairy tales) was immortal, strong, bony and lean (Figure 1). Was it his passion for the young princess Vassilisa, the Beautiful, who rendered him immortal? Did he lose his appetite because of his tragic love? Or was he secretly taking a rapalog such as rapamyin (Sirolimus), Temsirolimus, Everolimus and Deforolimus. And did Koschei benefit from benevolent glucose intolerance? Or, in contrast, was he insulin hypersensitive? Here are some answers and subsequent questions.

## Rapamycin Prevents Obesity

In mice on high-fat diet, rapamycin decreases obesity and prevents weight gain.^1, 2, 3, 4^ In rats, rapamycin (3 times per week) decreased age-associated weight gain.^5^ Also, chronic (everyday) treatment with rapamycin reduces adiposity and body weight.^6, 7^ (In some strains, chronic daily treatment was associated with insulin resistance (IR), but more on that latter). In humans, rapamycin decreases the size of fat cells (adipocytes) and body weight.^5^ In humans, kidney transplantation is associated with weight gain, which is preventable by rapamycin.^8^ So, at least at high doses, rapamycin can decrease weight gain in mice, rats and humans. Yet, Koschei was unusually skinny and there is no data that rapamycin can cause such a severe weight loss.

### How Rapamycin Prevents Obesity

- Rapamycin increases lypolysis, releasing fatty acids from the fat tissue.^9, 10, 11, 12^
- Rapamycin prevents entry of lipoproteins into the tissues.^6^
- Rapamycin decreases insulin secretion, therefore, preventing insulin-induced obesity.^13^
- Rapamycin prevents adipocyte differentiation.^10, 14, 15, 16^

Rapamycin increases lipolysis and decreases, this can lead to hyperlipidemia (see for explanation schema 2 in^17^). Hyperlipidemia (or dyslipidemia) is a biomarker of the treatment with high doses of rapamycin and evirolimus.^9^ Rapalog-induced dyslipidemia is a benevolent sign of therapeutic effects. In fact, rapamycin prevents atherosclerosis.^18, 19, 20^

Hyperlipidemia is rapidly reversible.^21^ Eventually, hyperlipidemia disappears despite chronic use of rapamycin.^22^ Noteworthy, hyperlipidemia can be diminished by lipid-lowering drugs, as shown in renal transplant patients who were receiving rapamycin.^23^

Fatty acids are burned by the muscles (especially during physical exercise) and also incorporated into lipoproteins by the liver.

We can hypothesize that benevolent dyslipidemia can be diminished by the following:

- Physical exercise (the muscle burns lipids).
- Calorie restriction.

These two predictions need to be tested.

### mTOR Causes Obesity and IR

Mammalian target of rapamycin (mTOR) is a nutrient-sensing pathway.^24, 25, 26, 27, 28, 29, 30, 31^ Nutrients such as glucose, amino and fatty acids activate mTOR and also increase insulin, which also activates mTOR. In the fat tissue, mTOR promotes adipocyte differentiation and hypertrophy, increases lipogenesis (synthesis of triglycerids) and decreases lipolysis (hydrolysis of triglycerides), leading to fat accumulation or obesity. In a vicious cycle, obesity activates mTOR.^32, 33^

To limit its overactivation, mTOR blocks insulin signaling, causing IR.^34, 35, 36, 37, 38, 39^ Rapamycin and calorie restriction (CR) can reverse IR.^32, 34, 40, 41, 42, 43, 44, 45, 46, 47, 48, 49^ For example, in healthy men, infusion of amino acids activates mTOR causing IR.^44, 46^ Administration of 6 mg rapamycin before amino acids prevents IR.^46^ Noteworthy, IR and metabolic syndrome are multifactorial.^50, 51, 52, 53, 54, 55, 56, 57, 58, 59, 60, 61^

## The Misunderstood Effect: Benevolent IR

The most common argument against rapamycin is that it causes IR. Somehow, this is the only rumor that many scientists heard about rapamycin. In fact, glucose intolerance and IR was observed in a few strains of rodents treated daily with high doses of rapamycin.^2, 62, 63, 64^ Yet, this was not detrimental for animal health. In contrast, IR was associated with weight loss and/or extended life span. Furthermore, unlike C57BL/6 mice,^64^ genetically heterogeneous HET3 mice on a rapamycin diet were glucose intolerant but insulin sensitive.^65^ Ironically, although believing that rapamycin is dangerous, most scientists do not know the difference between glucose intolerance and IR. They know even less about classic conditions of spectacular glucose intolerance and IR. Claude Bernard (19th century) described that during starvation humans and dogs develop reversible starvation-diabetes.^66^ If a starved animal (or human) consumes sugar, this sugar will appear in the urine, forcing water to follow (polyuria). The word ‘diabetes' means an increased amount of urine (polyuria). And ‘mellitus' means sweet. This sweet taste had been noticed in the urine by the ancient Greeks. So starvation is accompanied by the most definitive symptom of ‘diabetes mellitus'. This is a reversible condition to cope with starvation.

## Why Starvation Is Manifested by Benevolent Pseudodiabetes?

**Figure 2:**
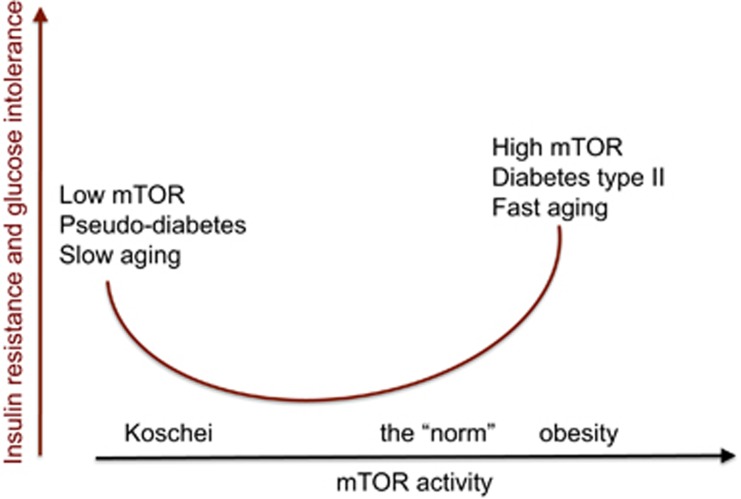
Insulin-resistance: two opposite conditions. Insulin resistance (IR) can be caused by the activation of mTOR and, paradoxically, by mTOR inhibition. In the first case, IR is detrimental for health, whereas in the second case it is benevolent^13, 66, 67^

During fasting, lipolysis is increased providing the ‘fuel' (free fatty acids and glycerol) for the peripheral tissues. The brain depends on glucose (and ketones). In the liver, amino acids are converted into glucose (gluconeogenesis) and fatty acid into ketones. To spare glucose for the brain, insulin secretion is inhibited and peripheral tissues become insulin resistant. Low insulin levels and IR are manifested as glucose intolerance: if a starved person consumes glucose, it is not metabolized by the tissues, its blood levels rose and glucose appears in the urine. Also, the liver produces ketones from lipids (to feed the brain). Production of ketones is a hallmark of type I diabetes. Starvation-induced pseudodiabetes is benevolent because they are associated with inhibited mTOR.^67^ In contrast, in the modern time, IR (as we know it) is associated with obesity and leads to diabetes type II.^40, 13^ This harmful IR is associated with over-activation of mTOR and aging (Figure 2).

## Calorie Restriction

CR extends life span in numerous species from worm to mammals.^11, 28, 68, 69, 70, 71, 72, 73, 74, 75, 76, 77, 78, 79, 80, 81^ CR prevents age-related diseases including cancer and sarcopenia.^82, 83, 84^ Whereas moderate CR increases insulin sensitivity, severe CR causes signs of IR.^85^ Among individuals who had been practicing severe CR, 40% of CR individuals showed ‘diabetic-like' glucose intolerance.^85^ In theory, starvation would be beneficial for health, but cannot last long enough for obvious reason – death from starvation. But high doses of rapamycin can mimic severe CR without actual nutrient deficiency, thus lacking harmful effects of starvation.

## Koschei Was not Starved

Definitely, Koschei was not starved. He was bonny and strong and this is not compatible with starvation. Fasting that is manifested by ‘diabetes' (sugar in the urine) cannot last too long to extend life span but rapamycin can. And since rapamycin does not decrease food consumption, it may extend life span dramatically, while moderately preventing obesity. Importantly, rapamycin increases skeletal muscle and bone mass.^86^ Given that Koschei was deathless, healthy, strong (muscular) and bony, he perhaps used CR-mimetic such as rapamycin, rather than severe CR.

## Rapamycin Plus Moderate CR

Because rapamycin inhibits mTOR but not food consumption, rapamycin is expected to disproportionally increase life span compared with its moderate effects on body weight. For example, at low doses and frequencies, which do not cause IR and other metabolic alterations, rapamycin still extends life span in mice.^87^ As we discussed, acute treatment by rapamycin increases insulin sensitivity. Pulse (intermittent) treatment with rapamycin (either once a week or every other week or intermittent short courses) extends life span,^88, 89, 90, 91, 92, 93^ while maintaining insulin sensitivity.^87^ In high-fat diet-fed C57BL/6 mice, weekly rapamycin for 22 weeks improved metabolic and immune status. Rapamycin-treated mice were leaner and were protected against IR and mTORC2 activity was intact.^4^ So, life extension by rapamycin can be associated with either IR or insulin sensitization depending on the dose and the frequency of administration. The life extension and anticancer effects were detectable at low-frequency administration, when little effect on weight was observed. Yet Koschei was extraordinary lean. We can consider two scenarios. First, he might use very high doses of rapamycin to develop ‘starvation-like diabetes', which can be followed by weight loss. (Note: weight loss is a symptom of type I diabetes). Although high chronic doses of rapamycin in some strains of mice cause IR, this IR did not reach the magnitude of full-blown ‘pseudodiabetes mellitus'. According to second scenario, Koschei combined rapamycin with standard CR (not starvation). We can expect that this will both extend life span and eliminate fat tissue. In agreement with second scenario, Koschei was known to be greedy, so CR was naturally added to rapamycin. And he should not experience diabetic-like polyurea because he did not eat sweets or sugar, but instead his diet consisted from small amount of meat (human), fish (mermaid) and fresh vegetables (nettle).

## Once Again on Benevolent IR

In contrast, starvation/rapamycin-induced IR is associated with inhibited mTOR (Figure 1). In all animal models, IR coupled with low mTOR is associated with health and life span extension.^67^ Is benevolent IR and pseudodiabetes a goal of rapamycin treatment for maximal life span extension? Or, in contrast, this should be avoided? In other words, should we use high doses of rapamycin daily or pulse (intermittent) treatment.

Apart from the question whether rapamycin-induced IR is benevolent or not, it is unclear what is its exact mechanism. In different studies, IR was accompanied either by low or high insulin levels. In some studies, IR was associated with low activation of Akt by insulin,^64, 94^ whereas in other studies rapamycin promoted IR despite normal activation of the Akt axis.^6^ In cell culture, rapamycin reverses IR caused by glucose and does not cause IR even at chronic (2 weeks) use.^95^

Thus, details of rapamycin-induced IR are still unclear. What is clear is that at both high and low doses, at chronic and intermittent administrations, rapamycin extends life- and health-span in mice. Also, it was taken by millions of humans in high doses daily, even though transplant and cancer patients were in bad health to start with. The most noticeable side effects of rapalogs (rapamycin, tecrlolimus, everolimus) are prevention of cancer^96, 97, 98^ and regression of heart hypertrophy in kidney transplant recipients.^99^ Rapalogs are anticancer drugs.^100, 101, 102, 103, 104, 105, 106, 107, 108, 109^

## Rapalogs as Anti-aging Drugs

Nutrients activate mTOR pathway, which drives cellular growth and functions, and then geroconversion and hyperfunctions.^110^ On organismal level, mTOR drives growth early in life and aging later in life.^111, 112^ Rapamycin slows aging and extends life span in mice.^113, 114, 115, 116, 117, 118, 119, 120, 121^ What is the cellular mechanism that allows rapamycin to slow organismal aging? Rapamycin slows down geroconversion: conversion from quiescence to irreversible senescence.^122, 123, 124, 125, 126, 127, 128, 129, 130^ Senescence is characterized by cellular hyperfunction (hyper secretion, hypertrophy, pro-inflammation and so on.^131, 132, 133, 134, 135, 136^ This cellular hyperfunction also cause a feedback signal resistance (such as IR) to limit hyperfunctions. A combination of hyperfunctions and signal resistance leads to alterations in homeostasis and initiates age-related diseases such as obesity, atherosclerosis, hypertension, neurodegeneration, osteoporosis, sarcopenia.^30, 119, 137, 138^ Cancer is preventable by rapamycin.^88, 90, 91, 96, 97, 98, 136, 139, 140, 141, 142, 143, 144, 145, 146^ Rapamycin prevents age-related diseases in rodents from macular degeneration and obesity to cancer and heart dysfunction.^142, 146, 147, 148, 149, 150, 151, 152^ Rapamycin also extends life span in normal and cancer-prone mice as well as in mice with premature aging syndromes.^93, 153^ In the latter case, rapamycin at an average extended life span more 100% and maximal survival >300%.^153^

## The Anti-aging Formula

Koschei was constantly fighting with enemies. So physical exercise was a part of his daily life. Mobilized by rapamycin, lipids can be burned by the muscle during physical exercise. By itself, chronic physical exercise inhibits mTOR and increases insulin sensitivity.^154^ Thus, rapamycin was combined with moderate CR (based on vegetables and fish) and physical exercise.

There are several clinically approved, widely used drugs that could be added to the rapamycin CR/exercise combination. They include metformin, aspirin, inhibitors of angiotensin II and propranolol.

It was shown almost 50 years ago that phenformin and metformin, anti-diabetic drugs that improve IR, also slow down aging and prevent cancer in rodents.^100, 106, 155, 156, 157, 158, 159, 160, 161, 162, 163^ These effects were explained from the mTOR perspective, revealing a rationale to combine rapamycin and metformin.^164^ Two agents may even cancel each other side effects. For example, whereas metformin can increase lactate production, rapamycin decreases it.^165^ Metformin also prevents cancer and other age-related diseases in humans.^166, 167, 168, 169, 170, 171, 172, 173, 174^

Aspirin, an anti-inflammatory agent, decreases pro-inflammation, a marker of senescence, as well as inhibits hyperfunctions of blood platelets and endothelial cells.^133, 175, 176^ There is increasing evidence that aspirin is beneficial in the prevention of multiple age-related diseases and their complications.^177, 178, 179, 180, 181, 182, 183, 184^ Aspirin increases life span of genetically heterogeneous male mice^179^ and even in the worm *Caenorhabditis elegans*.^185^

Angiotensin II activates mTOR pathway^186^ and is involved in aging and age-related diseases in mammals.^187, 188^ Disruption of the Ang II type 1 receptor promotes longevity in mice. At 29 months, when all wild-type animals died, 85% mice lacking the receptor were still alive. These remaining AT1−/− mice lived for an additional 7 months, with life span 26% longer than controls.^189^ Angiotensin II receptor blockers (ARB) (Valsartan, Telmisartan, Losartan) as well as angiotensin-converting enzyme inhibitors (Captopril, Lisinopril, Enalapril, Ramipril) are widely used as therapy for hypertension. Long-term angiotensin-converting enzyme inhibition or ARB doubles life span of hypertensive rats.^190, 191^ In healthy (normal blood pressure) rats, long-term enalapril treatment decreases body weight gain and prolonged life span.^192^ Long-term use of ARBs is associated with a lower incidence of cancer occurrence, thereby suggesting that ARBs may prevent cancer development.^193^

Propranolol, a non-selective beta-adrenergic blocker, is widely used to treat hypertension and ischemic heart disease. In addition, propranolol prevents cancer^194, 195, 196, 197^ and hepatic steatosis.^198^ Also, berberine and statins^199^ can be included into the anti-aging formula, especially given that statins prevent rapamycin-induced dyslipidemia.^23^

## Conclusion: Lessons Learned from Koschei

The creators of fairy tales noticed that the extraordinary longevity is associated with thinness, whereas obese people do not live long. It is not a coincidence that another character of Slavic tales, Baba Yaga the bony leg (kostianaia noga), was extremely old and thin. She cooked potion (зелье), an anti-aging mixture, for Koschei and herself. Now we can compose this mixture by using available drugs. The cornerstone of the formula is a rapalog such as rapamycin. Yet, gerontologists claim that rapamycin cannot be used in humans because of its terrible side effects. This modern tale about side effects of rapamycin might surprise physicians, who have prescribed rapamycin, everalimus to millions of patients worldwide. But practicing doctors do not read basic science papers. Why this misinformation circulates among gerontologists and other basic scientists. May be because Koschei and Baba Yaga were evil and had long curly hair (side effects). Or there are other reasons. I will discuss this in forthcoming article ‘Does mankind deserve rapamycin'.
